# Hypercalcemia in Patients After Kidney Transplantation

**DOI:** 10.7759/cureus.95057

**Published:** 2025-10-21

**Authors:** Miłosz Miedziaszczyk, Katarzyna Lacka, Aleksander Bajon, Dominik Lewandowski, Marta Kamalska, Piotr Zelga, Lukasz Swiatek, Marek Karczewski, Ilona Idasiak-Piechocka

**Affiliations:** 1 Department of Clinical Pharmacy and Biopharmacy, Poznan University of Medical Sciences, Poznan, POL; 2 Department of Endocrinology, Metabolism and Internal Medicine, Poznan University of Medical Sciences, Poznan, POL; 3 Student’s Research Group, Department of General and Transplant Surgery, Poznan University of Medical Sciences, Poznan, POL; 4 Department of General and Transplant Surgery, Poznan University of Medical Sciences, Poznan, POL

**Keywords:** calcium homeostasis, graft function, hypercalcemia, kidney transplantation, persistent hyperparathyroidism, tertiary hyperparathyroidism

## Abstract

Mineral and bone disorders are common complications of chronic kidney disease. Persistent hyperparathyroidism can develop after kidney transplantation (KTx), often within the first year, and may lead to hypercalcemia that can negatively impact graft function. This retrospective study aimed to assess the prevalence of hypercalcemia within the first three years following KTx and evaluate its impact on renal graft filtration function. Eighty-four patients (29 women, 55 men; mean age 53 ± 13 years) were included. Total calcium and creatinine levels were measured during the first, second, and third year post-transplantation. Statistical analyses were performed using the MedCalc software (MedCalc Software Ltd., Ostend, Belgium). Hypercalcemia was observed in 16 (19.1%) patients at one year, 14 (16.7%) at two years, and 17 (20.2%) at three years post-transplantation. A gradual and statistically significant increase in total and ionized calcium levels was noted over three years. No significant correlation was found between ionized calcium and serum creatinine or estimated glomerular filtration rate (eGFR) in any period analyzed. Hypercalcemia is a common finding after KTx, with increasing calcium levels observed over time. Although no significant association with graft function was identified, further long-term studies are warranted. Routine laboratory monitoring for hypercalcemia and hyperparathyroidism in kidney transplant recipients is recommended, in accordance with KDIGO guidelines.

## Introduction

A significant number of kidney transplant recipients develop hypercalcemia. In most cases, elevated serum calcium levels are associated with persistent hyperparathyroidism [1-3]. Consequently, some patients require parathyroidectomy to control parathyroid hormone (PTH) levels and achieve a satisfactory calcium balance. An alternative therapeutic approach involves the use of calcimimetic agents such as cinacalcet, which help regulate calcium-phosphorus homeostasis and have a positive effect on bone metabolism in transplant recipients [4].

However, hypercalcemia may also result from other causes, including malignancies such as multiple myeloma and lymphomas, as well as systemic diseases like sarcoidosis or infectious granulomatous conditions (e.g., tuberculosis and fungal infections). Interestingly, a link between hypercalcemia and *Pneumocystis jirovecii* pneumonia has been reported in immunocompromised patients [5]. Iatrogenic hypercalcemia should also be considered, as excessive intake of vitamin D3 (cholecalciferol) or calcium supplements has become more common with the widespread use of over-the-counter preparations that vary considerably in composition and dosage. Thus, overuse of such supplements also needs to be accounted for [6,7].

The clinical manifestations of hypercalcemia in the general population range from mild, such as anorexia, nausea, constipation, polyuria, and polydipsia, to more severe symptoms, including cognitive impairment, drowsiness, and obtundation [8]. Electrolyte imbalance also contributes to progressive changes in bone health. The most common bone morphology alteration is low bone turnover disease, although the incidence of high-turnover osteopathy tends to decrease after transplantation. Moreover, disorders such as fibrous osteitis and adynamic bone disease can lead to decreased bone mineral density and increased fracture risk. Evidence regarding the effect of persistent hypercalcemia on graft function remains inconsistent. Some studies suggest that sustained hypercalcemia after transplantation may contribute to the development of chronic allograft nephropathy [9] through tubulointerstitial calcification [10]. In contrast, Kim et al. [3] reported no adverse impact of hypercalcemia on graft outcomes. The objective of this retrospective study was to assess the prevalence of hypercalcemia in kidney transplant patients within the first three years following transplantation and to evaluate its impact on renal graft filtration function in this population.

## Materials and methods

Study design

This retrospective study included 84 patients (29 women and 55 men) with a mean age of 53 ± 13 years. Access to anonymized data for research purposes was obtained on May 29, 2023. The authors did not have access to any information that could identify individual participants during or after data collection. The inclusion criteria comprised measurements of total calcium and creatinine levels within the first three years following kidney transplantation (KTx). Additional parameters tested were: ionized calcium, inorganic phosphorus, PTH, GFR. Changes in these parameters were analyzed as a three-year progression within the same cohort of patients. Exclusion criteria included kidney transplant rejection, history of parathyroidectomy, use of thiazide diuretics, and diagnosis of parathyroid adenoma. The glomerular filtration rate (GFR) was calculated for each patient using the Chronic Kidney Disease Epidemiology Collaboration (CKD-EPI) creatinine equation. The clinical and demographic characteristics of the study population are summarized in Table 1. Anemia was defined as the presence of at least two decreased parameters among the following: hemoglobin <13.0 g/dL in men or <12.0 g/dL in women; hematocrit <40% in men or <37% in women; and red blood cell count <4.2 million/mm³ in men or <3.5 million/mm³ in women. All study procedures were conducted in accordance with the principles of the Declaration of Helsinki.

**Table 1:** Characteristics of the study population. Data are expressed as number of patients (n) and as mean ± standard deviation (SD).

	Baseline	Follow-up	
Time after kidney transplantation (KTx)	One year	Two years	Three years
Women (n = 29), age (years)	51.2 ± 13.4	52.2 ± 13.4	53.2 ± 13.4
Men (n = 55), age (years)	50.6 ± 12.7	51.6 ± 12.7	52.6 ± 12.7
Hypercalcemia defined as total serum calcium level above 10.5 mg/dL, n	16	14	17
Total calcium concentration (mg/dL)	9.94 ± 0.65	10.02 ± 0.55	10.06 ± 0.59
Ionized calcium concentration (mg/dL)	5.41 ± 0.38	5.50 ± 0.35	5.52 ± 0.37
Inorganic phosphorus concentration (mg/dL)	3.26 ± 1.12	3.10 ± 0.82	3.38 ± 0.93
Parathyroid hormone concentration > 65 pg/mL, n	75	76	67
Creatinine concentration (mg/dL)	1.57 ± 0.65	1.56 ± 0.48	1.60 ± 0.53
Glomerular filtration rate (GFR) (mL/min/1.73 m^2^)	51.0 ± 18.3	49.4 ± 17.6	48.8 ± 19.3
Glomerular filtration rate (GFR) < 60 mL/min/1.73 m^2^, n	71	69	71
Hemoglobin (g/dL)	13.4 ± 1.9	13.5 ± 1.9	13.7 ± 1.7
Hematocrit (%)	40.4 ± 5.7	40.6 ± 6.0	41.2 ± 4.8
Red blood cells (million/mm^3)^	4.67 ± 0.66	4.71 ± 0.67	4.73 ± 0.61
Anemia, n	30	23	31

Statistical analysis

Statistical calculations were performed using MedCalc software (MedCalc Software Ltd., Ostend, Belgium). The Shapiro-Wilk test was used to assess whether calcium, creatinine, and eGFR values followed a normal distribution. Differences between variables with normal distribution were analyzed using the t-test, while those with non-normal distribution were analyzed using the Wilcoxon signed-rank test. For unpaired variables with normal distribution and equal variances, the t-test was used; in cases of unequal variances, the Welch test was applied. Unpaired variables with non-normal distribution were compared using the Mann-Whitney U test. Correlations between two non-normally distributed variables were assessed using Spearman’s rank correlation.

## Results

Parameter changes during the three-year period following kidney transplantation

The results of calcium, parathyroid hormone, creatinine concentration, and eGFR changes over the three-year period after KTx are presented in Table 2. 

**Table 2:** Parameter changes over time. This table compares the values obtained one, two, and three years after kidney transplantation (KTx). Data are presented as mean ± standard deviation (SD). A p-value of less than 0.05 is considered statistically significant. The differences between the variables with normal distribution were calculated using the t-test (^). The differences between the variables with non-normal distribution were calculated using the Wilcoxon signed-rank test (*).

Parameter	Baseline (1 year after KTx)	After X time	Shapiro-Wilk test (p-value)	95% Cl	p-value
C_total calcium_ (mg/dL) after 2 years	9.94 ± 0.65	10.02 ± 0.55	0.0001	-	0.1161*
C_total calcium_ (mg/dL) after 3 years	9.94 ± 0.65	10.06 ± 0.59	0.0019	-	0.0154*
C_ionized calcium_ (mg/dL) after 2 years	5.41 ± 0.38	5.50 ± 0.35	0.6533	0.0272 to 0.1576	0.0067^
C_ionized calcium_ (mg/dL) after 3 years	5.41 ± 0.38	5.52 ± 0.37	0.3042	0.0345 to 0.1902	0.0059^
C_parathyroid hormone_ (pg/mL) after 2 years	135.63 ± 56.05	122.73 ± 71.72	0.9576	-25.3423 to 17.3962	0.6925^
C_parathyroid hormone_ (pg/mL) after 3 years	135.63 ± 56.05	133.12 ± 107.00	0.0001	-	0.9323*
C_creatinine_ (mg/dL) after 2 years	1.57 ± 0.65	1.56 ± 0.48	0.0001	-	0.5560*
C_creatinine_ (mg/dL) after 3 years	1.57 ± 0.65	1.60 ± 0.53	0.0001	-	0.3554*
GFR (mL/min/1.73 m^2^) after 2 years	51.0 ± 18.3	49.4 ± 17.6	0.5656	-4.0320 to 0.8892	0.2076^
GFR (mL/min/1.73 m^2^) after 3 years	51.0 ± 18.3	48.8 ± 19.3	0.0400	-	0.1968*

**Figure 1:**
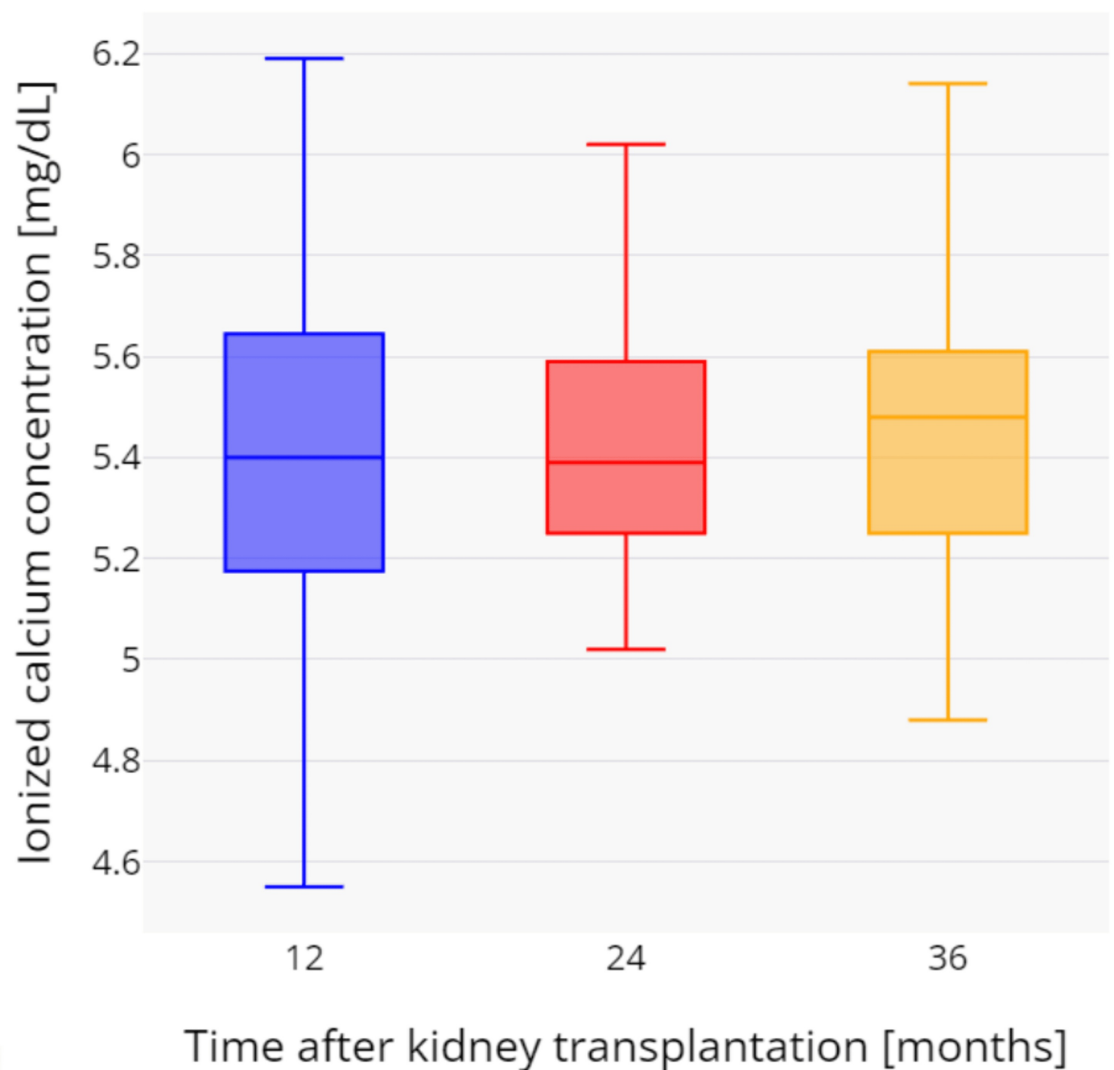
Changes in ionized calcium concentrations during the three-year period after kidney transplantation.

**Figure 2:**
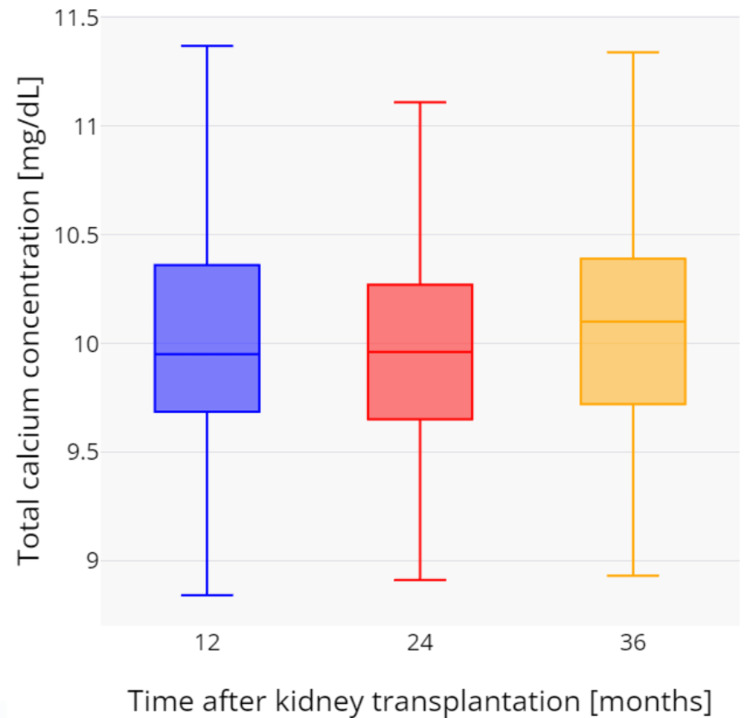
Changes in total calcium concentrations during the three-year period after kidney transplantation.

During the three-year observation period, a gradual and statistically significant increase in total serum calcium was observed. The mean total calcium values were as follows: after one year, 9.94 ± 0.65 mg/dL; after two years, 10.02 ± 0.55 mg/dL (p = 0.1161); and after three years, 10.06 ± 0.59 mg/dL (p = 0.0154). Similarly, ionized calcium concentration increased over time, with the following mean values: after one year, 5.41 ± 0.38 mg/dL; after two years, 5.50 ± 0.35 mg/dL (p = 0.0067); and after three years, 5.52 ± 0.37 mg/dL (p = 0.0059). Hypercalcemia, defined as total serum calcium concentration above 10.5 mg/dL, was observed in 16 (19.1%) patients after one year, 14 (16.7%) after two years, and 17 (20.2%) after three years following kidney transplantation. All hypercalcemia cases were new-onset, except for 12 patients who had persistently elevated calcium levels throughout the study period. Changes in total and ionized calcium concentrations over time are presented in Figure 1 and Figure 2. Mean serum creatinine levels were 1.58 ± 0.65 mg/dL after one year, 1.56 ± 0.49 mg/dL after two years, and 1.59 ± 0.54 mg/dL after three years. There was no significant correlation between creatinine and ionized calcium at any time point: 12 months (rho = -0.144, p = 0.3815), 24 months (rho = -0.217, p = 0.1914), and 36 months (rho = -0.234, p = 0.1094). Mean eGFR values were 51.0 ± 18.3, 49.4 ± 17.6, and 48.8 ± 19.3 mL/min/1.73 m² after one, two, and three years, respectively. Similarly, there was no significant correlation between eGFR and ionized calcium at any time point: 12 months (rho = 0.187, p = 0.2472), 24 months (rho = 0.254, p = 0.1357), and 36 months (rho = 0.243, p = 0.0999).

Immunosuppressive regimen

Forty-three patients (51.2%) received a standard immunosuppressive regimen consisting of tacrolimus (TAC), mycophenolate mofetil (MM), and glucocorticosteroids (GKS; prednisone or methylprednisolone). TAC combined with MM was administered in 8 patients (9.5%), while 11 patients (13.1%) received cyclosporine, MM, and GKS. Four patients (4.8%) were treated with cyclosporine and MM. The remaining 18 patients received alternative immunosuppressive regimens.

Impact of medications affecting calcium homeostasis 

Thirty patients (35.7%) received cholecalciferol supplementation. In 11 patients (13.1%), 1α-hydroxycholecalciferol (alfacalcidol) was administered, while 10 patients (11.9%) received calcium ion supplementation in the form of calcium carbonate.

PTH concentrations were compared between patients who did not receive supplementation and those who did. After one year, the mean PTH levels were as follows: calcium ions (155.6 ± 68.7 vs. 117.0 ± 76.2 pg/mL, p = 0.3603), cholecalciferol (155.6 ± 68.7 vs. 108.2 ± 51.1 pg/mL, p = 0.0437), and alfacalcidol (155.6 ± 68.7 vs. 129.6 ± 67.5 pg/mL, p = 0.4975). After two years, the respective comparisons were: calcium ions (173.5 ± 77.6 vs. 130.6 ± 105.3 pg/mL, p = 0.3017), cholecalciferol (173.5 ± 77.6 vs. 111.2 ± 51.7 pg/mL, p = 0.0140), and alfacalcidol (173.5 ± 77.6 vs. 113.0 ± 80.4 pg/mL, p = 0.2417). After three years, PTH levels were: calcium ions (137.5 ± 89.1 vs. 149.2 ± 65.2 pg/mL, p = 0.3831), cholecalciferol (137.5 ± 89.1 vs. 109.7 ± 47.5 pg/mL, p = 0.4144), and alfacalcidol (137.5 ± 89.1 vs. 112.4 ± 69.5 pg/mL, p = 0.3923).

Ionized calcium concentrations were also compared between the same groups. One year after KTx, mean levels were: calcium ions (5.42 ± 0.43 vs. 5.52 ± 0.40 mg/dL, p = 0.6737), cholecalciferol (5.42 ± 0.43 vs. 6.36 ± 2.07 mg/dL, p = 0.3965), and alfacalcidol (5.42 ± 0.43 vs. 5.44 ± 0.33 mg/dL, p = 0.9121). After two years, values were: calcium ions (5.58 ± 0.38 vs. 5.45 ± 0.51 mg/dL, p = 0.6508), cholecalciferol (5.58 ± 0.38 vs. 5.48 ± 0.34 mg/dL, p = 0.2932), and alfacalcidol (5.58 ± 0.38 vs. 6.66 ± 2.50 mg/dL, p = 0.6546). After three years, corresponding values were: calcium ions (5.56 ± 0.37 vs. 5.43 ± 0.44 mg/dL, p = 0.4464), cholecalciferol (5.56 ± 0.37 vs. 5.51 ± 0.29 mg/dL, p = 0.5806), and alfacalcidol (5.56 ± 0.37 vs. 6.09 ± 1.95 mg/dL, p = 0.8531).

Comparison of patients with and without hypercalcemia

Table 3 presents the characteristics of patients with and without hypercalcemia.

**Table 3:** Characteristics of the patients with and without hypercalcemia. Data are presented as mean ± standard deviation (SD) or as percentage (%). A p-value of less than 0.05 is considered statistically significant. The differences between the unpaired variables with normal distribution and equal variances were calculated using the t-test (^), whereas the Welch test was applied in the case of different variances. The differences between the unpaired variables with non-normal distribution were calculated using the Mann-Whitney U test (*).

Time after KTx	One year after KTx		p-value	Two years after KTx		p-value	Three years after KTx		p-value
Group	Without hypercalcemia (n = 68)	With hypercalcemia (n = 16)		Without hypercalcemia (n = 70)	With hypercalcemia (n = 14)		Without hypercalcemia (n = 67)	With hypercalcemia (n = 17)	
Total calcium concentration (mg/dL)	9.75 ± 0.52	10.81 ± 0.24	0.0001*	9.85 ± 0.40	10.96 ± 0.26	0.0001^	9.84 ± 0.41	10.84 ± 0.29	0.0001*
Ionized calcium concentration (mg/dL)	5.31 ± 0.31	5.77 ± 0.29	0.0002^	5.44 ± 0.30	6.02 ± 0.39	0.0025*	5.62 ± 0.95	6.35 ± 1.59	0.0007*
Inorganic phosphorus concentration (mg/dL)	3.50 ± 1.18	2.50 ± 0.34	0.0230*	3.24 ± 0.81	2.57 ± 0.73	0.0593*	3.29 ± 0.71	2.76 ± 0.19	0.1512^
Parathyroid hormone concentration (pg/mL)	125.5 ± 50.1	159.3 ± 66.7	0.2252^	115.3 ± 75.7	143.7 ± 60.0	0.6576*	115.9 ± 109.8	184.6 ± 81.4	0.0006*
Parathyroid hormone concentration > 65 pg/mL (%)	89.29	100		90.48	100		79.76	100	
Creatinine concentration (mg/dL)	1.62 ± 0.68	1.36 ± 0.44	0.0711^	1.60 ± 0.50	1.29 ± 0.30	0.0301*	1.60 ± 0.53	1.54 ± 0.48	0.6861*
Glomerular filtration rate (GFR)(mL/min/1.73 m^2^)	49.4 ± 18.1	58.3 ± 18.4	0.4248^	47.7 ± 17.6	61.0 ± 13.7	0.0049*	36.9 ± 25.6	52.1 ± 21.8	0.0279*
Glomerular filtration rate (GFR) < 60 mL/min/1.73 m^2 ^(%)	73.81	68.75		80.95	57.14		77.38	64.71	
Cholecalciferol supplementation (%)	35.7	56.25		35.7	57.14		35.7	41.12	
1α-hydroxycholecalciferol supplementation (%)	13.1	12.5		13.1	14.29		13.1	17.65	
Calcium carbonate supplementation (%)	11.9	6.25		11.9	7.14		11.9	11.76	

## Discussion

In our retrospective study, hypercalcemia was observed in 16 patients (19.1%), 14 patients (16.7%), and 17 patients (20.2%) at one, two, and three years following KTx, respectively. According to literature data, the reported incidence of hypercalcemia two years after transplantation ranges from 1.4% to 47% (median 10.0%) [2,11,12]. The wide variability in these figures may be attributed to differences in calcium concentration cut-off values, therapeutic approaches to hypercalcemia, patient population characteristics, and the duration of follow-up across studies.

In our study, we observed a gradual increase in calcium concentrations (although the differences were small) over the three-year period: 9.94 ± 0.65 vs. 10.06 ± 0.59 mg/dL (p = 0.0154) for total calcium and 5.41 ± 0.38 vs. 5.52 ± 0.37 mg/dL (p = 0.0059) for ionized calcium. Muirhead et al., in a cohort of 1,000 patients, demonstrated a decreasing trend in calcium concentrations during the subsequent years (1-4) after KTx, with a corresponding reduction in the risk of hypercalcemia within 1-3 years post-transplant [2]. In contrast, Van de Cauter et al., studying 49 patients, reported that calcium concentrations remained unchanged in the years following transplantation [12]. Nonetheless, all authors emphasize the clinical significance of hypercalcemia in this patient population.

The main factor determining hypercalcemia in this group of patients is persistent hyperparathyroidism [1,2,9]. In our study, elevated PTH levels were found in 75 patients (89.3%), 76 patients (90.5%), and 67 patients (79.8%) at one, two, and three years following KTx, respectively. However, other specific factors affecting calcium metabolism should also be considered. Transplant recipients are immunosuppressed, which increases the risk of opportunistic infections such as tuberculosis, histoplasmosis, or fungal infections. Moreover, systemic diseases, including sarcoidosis and malignancies (such as multiple myeloma, lymphomas, and paraneoplastic syndromes), may also contribute to hypercalcemia. In addition, iatrogenic causes, for instance, excessive intake of over-the-counter preparations containing cholecalciferol or calcium supplements, should always be ruled out [1,6,7].

In the studied population, 41 patients (48%) received supplementation with cholecalciferol or its analog (alfacalcidol), while calcium supplements were administered in 10 cases (12%). The active form of vitamin D regulates PTH levels in the blood. These agents have a beneficial effect on bone mineralization and help prevent osteoporosis. Moreover, low levels of 1,25-dihydroxyvitamin D have been identified as a predictor of transplant rejection [13-15].

In our study, lower PTH concentrations were observed in the group receiving cholecalciferol supplementation compared with patients not taking vitamin D preparations during the three years following KTx (p = 0.0437, p = 0.0140, and p = 0.4144, respectively). These findings are consistent with data reported in previous studies [16,17]. However, the potential risk of hypercalcemia argues against the use of these agents in patients with calcium levels above the upper limit of normal [15,18,19]. According to the KDIGO 2017 guidelines [20], treatment with cholecalciferol, calcitriol/alfacalcidol, and/or antiresorptive agents may be considered within the first year after KTx in patients with an eGFR greater than approximately 30 mL/min/1.73 m² and low bone mineral density. Nonetheless, treatment decisions should be guided by the presence of chronic kidney disease-mineral and bone disorder (CKD-MBD), as indicated by abnormal calcium, phosphate, PTH, alkaline phosphatase, or calcifediol levels. In patients with hypercalcemia, the use of calcitriol or other vitamin D sterols should be reduced or discontinued.

Our results did not show a significant correlation between creatinine and total or ionized calcium in any of the analyzed periods, which is consistent with previous studies that also found no relationship between eGFR and hypercalcemia [2,3]. It is important to emphasize that we observed significant differences in renal filtration function two and three years after kidney transplantation. Patients with hypercalcemia had higher eGFR values than those without hypercalcemia. However, differences in baseline eGFR values should be considered, as they are influenced by multiple variables, and this interpretation should be made with caution. The literature data on the impact of hypercalcemia on renal function in kidney transplant recipients remain inconclusive. Allograft tubulointerstitial calcification has been reported to be more common and more progressive in patients with persistent hypercalcemia, whereas normalization of calcium levels may lead to regression of calcifications in some cases [10]. Some studies have also reported adverse effects on the transplanted kidney, such as increased serum creatinine or the development of tubulointerstitial calcifications [9,10]. The overlapping effects of various factors, including calcineurin inhibitor-induced nephrotoxicity, systemic diseases, and immune-mediated injury to the graft, can impair renal filtration and may contribute to discrepancies among study findings.

Histological findings indicate that tubulointerstitial calcifications are hallmarks of chronic transplant injury caused by prolonged hypercalcemia. Other contributing factors include donor-related factors (such as early arterionephrosclerosis), ischemia-reperfusion-related factors (such as cold ischemia time and the advantage of living donation), and immunologic factors (such as early rejection), all of which can affect kidney transplant function [10,21]. Clinical manifestations of hypercalcemia include nephrolithiasis, renal tubular acidosis, and systemic symptoms such as anorexia, constipation, abdominal pain, diabetes insipidus, anxiety, depression, headache, cognitive disturbances, lethargy, and muscle weakness [21].

The relationship between hypercalcemia and hyperparathyroidism primarily underscores the need to address the underlying cause [1,2,9]. The treatment of hyperparathyroidism includes paricalcitol, cinacalcet, or parathyroidectomy, all of which lower PTH levels [22]. However, according to previous analyses [23], only cinacalcet and parathyroidectomy were effective in reducing calcium levels in renal transplant patients with hypercalcemia. In contrast, paricalcitol was associated with increased serum calcium concentrations and is therefore not recommended for such cases.

The limitations of our study include the lack of data from the dialysis period, which prevented comparison of calcimimetic use before KTx between patients with and without hypercalcemia. Additionally, the study did not assess potential complications of hypercalcemia, which the authors plan to investigate in a future prospective study. The three-year observation period also limits the evaluation of the long-term effects of hypercalcemia on allograft function. Finally, the relatively small sample size (n = 84) and single-center design represent further limitations.

## Conclusions

The results of our study show that hypercalcemia is a common complication in kidney transplant patients. Both ionized and total calcium concentrations increased over the three years following transplantation. Persistent hyperparathyroidism appears to be one of the main causes of hypercalcemia. Although no significant association between hypercalcemia and kidney transplant function was observed during the study period, we believe that longer follow-up is needed to fully assess its impact. Based on these findings, we recommend regular laboratory testing in kidney transplant recipients to detect hypercalcemia and hyperparathyroidism in accordance with the KDIGO guidelines. In the immediate post-transplant period, serum calcium and phosphate should be measured at least weekly until levels stabilize. Beyond this period, the frequency of monitoring should depend on the presence and severity of abnormalities, as well as the rate of CKD progression. Specifically, for CKD G1T-G3bT, serum calcium and phosphate should be measured every 6-12 months, and PTH should be assessed once initially, with subsequent intervals determined by baseline values and CKD progression. In CKD G1T, calcium and phosphate levels should be monitored every 6-12 months, and PTH at least annually. Further prospective studies with longer observation periods are warranted to evaluate the long-term effects and potential complications of hypercalcemia in kidney transplant patients.
